# The evolution of health literacy assessment tools: a systematic review

**DOI:** 10.1186/1471-2458-14-1207

**Published:** 2014-11-24

**Authors:** Sibel Vildan Altin, Isabelle Finke, Sibylle Kautz-Freimuth, Stephanie Stock

**Affiliations:** Institute for Health Economics and Clinical Epidemiology, University Hospital of Cologne, Gleuelerstr 176-178, 50935 Cologne, Germany; Institute of Medical Biometry, Epidemiology and computer science (IMBEI) Medical Faculty of the Johannes Gutenberg-University Mainz, Obere Zahlbacher Str. 69, 55131 Mainz, Germany

**Keywords:** Health literacy, Measurement, Assessment tool, Reporting quality

## Abstract

**Background:**

Health literacy (HL) is seen as an increasingly relevant issue for global public health and requires a reliable and comprehensive operationalization. By now, there is limited evidence on how the development of tools measuring HL proceeded in recent years and if scholars considered existing methodological guidance when developing an instrument.

**Methods:**

We performed a systematic review of generic measurement tools developed to assess HL by searching PubMed, ERIC, CINAHL and Web of Knowledge (2009 forward). Two reviewers independently reviewed abstracts/ full text articles for inclusion according to predefined criteria. Additionally we conducted a reporting quality appraisal according to the survey reporting guideline SURGE.

**Results:**

We identified 17 articles reporting on the development and validation of 17 instruments measuring health literacy. More than two thirds of all instruments are based on a multidimensional construct of health literacy. Moreover, there is a trend towards a mixed measurement (self-report and direct test) of health literacy with 41% of instruments applying it, though results strongly indicate a weakness of coherence between the underlying constructs measured. Overall, almost every third instrument is based on assessment formats modeled on already existing functional literacy screeners such as the REALM or the TOFHLA and 30% of the included articles do not report on significant reporting features specified in the SURGE guideline.

**Conclusions:**

Scholars recently developing instruments that measure health literacy mainly comply with recommendations of the academic circle by applying multidimensional constructs and mixing up measurement approaches to capture health literacy comprehensively. Nonetheless, there is still a dependence on assessment formats, rooted in functional literacy measurement contradicting the widespread call for new instruments. All things considered, there is no clear “consensus” on HL measurement but a convergence to more comprehensive tools. Giving attention to this finding can help to offer direction towards the development of comparable and reliable health literacy assessment tools that effectively respond to the informational needs of populations.

**Electronic supplementary material:**

The online version of this article (doi:10.1186/1471-2458-14-1207) contains supplementary material, which is available to authorized users.

## Background

Health literacy is an important determinant of public and individual health and is seen as a core element of patient centered care [1]. In recent years there is a growing effort to adjust the structures of heath care systems according to the population’s health literacy to help them navigate through the layers of the health care system successfully [2]. The underlying objective is to enhance access to health care services for vulnerable populations [3].

Overall health literacy denotes “people’s knowledge, motivation and competences to access, understand, appraise and apply health information in order to make judgments and take decisions in everyday life concerning health care to maintain or improve quality of life during the life course” [4]. Improving people’s knowledge is of importance since there is a distinct interplay between limited health literacy and poor health outcomes as well as avoidable health care service utilization demonstrated in numerous studies [5–7]. Meanwhile the prevalence of limited health literacy is high, accounting for 26% of the population in the United States and between 29% and 62% among the populations of eight European countries [8, 9]. Consequently, the importance of health literacy has been recognized on a national and international level and great efforts are made to reduce the risk of limited health literacy by setting up international collaborations, national priority action plans and determining legal regulations [10, 11]. Following this course, the main key to mediate the transformation process to a health literacy friendly health care system is the availability of detailed and comparable information of population based health literacy [12].

Therefore the call for action regarding the development of an internationally comparable and reliable population based measure of health literacy is increasing [12].

By now there are several definitions and theoretical frameworks of health literacy in place serving as a foundation to operationalize health literacy by developing framework based measures [4]. These instruments have been developed to measure health literacy on the basis of skills related to finding, understanding, evaluating, communicating and using health related information in healthcare decision making [13, 14]. While using objective or subjective measurement modes by deriving a direct test of skills or obtaining a self-report of perceived skills, scholars identified central pillars of health literacy such as print, prose and document literacy, numeracy and oral literacy [15]. Though multiple measurement modes are applied, a number of specific critiques are traceable in the academic literature principally scrutinizing varying definitions and frameworks of health literacy as well as incomprehensive measurement approaches and inconsistent reporting of psychometric properties [16, 17]. Thus, health literacy involves a “constellation of skills” [18] including the ability to interpret documents, read and write prose (print literacy), use quantitative information (numeracy or quantitative literacy) as well as being able to communicate effectively (oral literacy) and all skills need to be addressed when developing a tool [15]. By now, there is no evidence on how health literacy measurement proceeded in the last few years and if recently published articles dealing with the development of health literacy measures consider the methodological critiques and recommendations of the academic circle that requires a set of features an instrument has to cover [16, 17].

In this systematic review, we evaluate the status quo of health literacy measurement by providing insights in the currently applied measurement approaches and modes. Further, we appraise the reporting quality of publications dealing with the development and validation of instruments measuring health literacy. The review will help to verify if currently developed tools aiming to measure health literacy consider methodological critiques in the academic literature and contribute to the improvement of health literacy measurement.

## Methods

**Figure 1:**
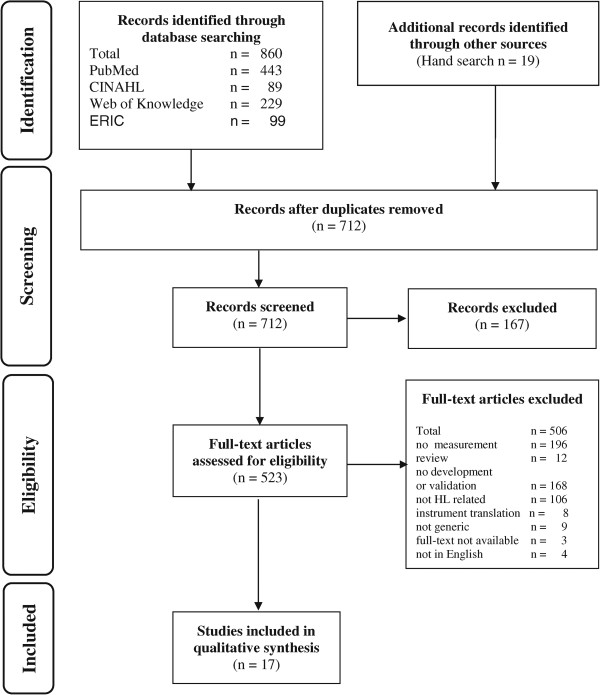
**PRISMA flow diagram of systematic review inclusion and exclusion process.**

We conducted a systematic review of generic measurement instruments developed and validated to assess health literacy. Our review is in accordance with the recently extended guidelines of the PRISMA statement for reporting systematic reviews [19] (see Figure 1 and Additional file 1). The used 27 item instrument ensures the transparent and complete reporting of systematic reviews and meta-analyses.

### Data sources and selection

The review was completed by using PubMed, the Educational Resources Information Center (ERIC), the Cumulative Index to Nursing and Allied Health Literature (CINAHL) and Web of Knowledge databases. Additionally references in already published reviews and other publications were screened and a manual search on websites and print sources dealing with health literacy measurement was conducted. The search strategies encompassed key words as well as MESH terms depending on the database and were supplemented by synonyms and thesaurus terms as described in Additional file 2. The search was performed from January 2009 to 24^th^ April, 2013 and was limited to fully available English language publications developing and validating (testing, evaluating) generic instruments to measure health literacy. We decided on the specific period of time to cover literature left out in previous reviews on health literacy measurement [13, 20]. The search was limited to instruments targeting adolescents and adults. Translations of instruments originally published before the search period were excluded.

### Data extraction and assessment of reporting quality

Articles were included by screening titles and abstracts of all unique publications and formulating full-text reports of all records passing the title/abstract screen by two independent reviewers. All health literacy instruments were categorized according to their characteristics including their purpose, applied health literacy taxonomy and construct, instrument design, availability, scoring method, validation-study sample characteristics as well as psychometric properties such as reliability and validity of the instrument. Additionally, a quality assessment according to the specifications of the reporting guidelines for survey research (SURGE) was performed. It encompasses reporting items in eight subdomains: article background, methods used, sample selection criteria, research tool characteristics, response rate as well as presentation of results, interpretation and discussions of findings and requirements of ethics and disclosure [21]. Categories within the framework were adapted when relevant for the appraisal of health literacy indices. The accurate reporting on the development and validation of instruments assessing patient reported outcomes such as health literacy is important in terms of an objective assessment of applied methods and identified psychometric properties of instruments and therefore the generalizability of study results. Especially during the research process transparency concerning methodological issues of indices can help to enhance overall study quality by allowing refinements on the instrument. The SURGE is an adequate instrument to appraise the reporting quality in surveys including detailed information on the characteristics of the used survey instruments. Therefore, it served as an appropriate instrument to appraise the reporting quality of health literacy indices.

After extracting the instrument characteristics these were entered into an evidence table and critically assessed for reporting quality by two independent raters, followed by a third rater checking the extracted information for accuracy. Disagreements were resolved by a consensus process between the three raters.

## Results

The PRISMA flow chart in Figure 1 summarises the results of the search process. Our search yielded 17 generic instruments. The majority of excluded articles were not measuring health literacy (n = 196) or did not report on the development or/and validation of a novel health literacy assessment tool (n = 168). Nine instruments had to be excluded due to a non-generic measurement approach [22–30] and eight were direct translations of already developed instruments [22, 28, 30–35] in several languages.

### Study characteristics

Among the 17 included publications on the development/validation of a generic health literacy measurement tools certain patterns can be identified. As depicted in Table 1, about one third of the instruments use either a direct test of an individual’s abilities (objective measurement) or the elicitation of self-reported abilities (subjective measurement). In studies using the objective measurement approach, patient abilities are assessed by solving tasks dealing with print literacy, numeracy or oral literacy whereas the self-report approach is characterized by the self-report of perceived abilities in multiple domains. Moreover according to Table 1 the combination of both measurement modes can be found in 41,2% of all identified instruments, respectively.

**Table 1:** **Measurement modes and approaches of health literacy**

	Number of generic instruments (n = 17)	
	N	%
All generic instruments	17	-
*Measurement modes*		
Print literacy	17	100
Oral literacy	3	17,6
Numeracy	8	47,1
Multidimensional measurement	16	94,1
*Measurement approaches*		
Objective measurement	5	29,4
Subjective measurement	5	29,4
Mixed measurement	7	41,2
Multidimensional construct	13	76,5

The generic instruments identified here consider the multi-dimensional measurement approach by applying print literacy in all instruments and measuring quantitative abilities in almost half of the identified tools. In contrast, only three instruments are considering communication skills of individuals when measuring an individual’s health literacy. Following the theoretical framework of health literacy being a multidimensional, dynamic construct [36] with an comprehensive approach, 76,5% of all identified instruments are based on a multidimensional construct of health literacy as shown in Table 1. Therefore multiple domains of health literacy are addressed such as healthcare information seeking, communication in the patient-provider encounter, interaction with the health care system and awareness of rights and responsibilities [37]. Additionally the utilization of a multidimensional measurement approach is pursued in almost all instruments mostly assessing print and quantitative literacy.

### Health literacy assessment by an objective measurement approach

The direct testing of competencies related to the health literacy construct is used frequently in the academic literature and five novel instruments were published in the search period. The Medical Term Recognition Test *(METER)* developed in the United States is a brief self-administered screening tool (2 min administration time) for the clinical setting and includes 40 medical words and 40 words without an actual meaning (non-words) while aiming the identification of the medical words [38]. The format of the tool includes many words from the Rapid Estimate of Adult Literacy in Medicine *(REALM)*[39]. Thus, there is a high correlation (r = 0.74) between the instruments [38]. The Short Assessment of Health Literacy in Spanish and English populations *(SAHL-S&E)* also uses a word recognition approach as applied in the REALM and combines these with a comprehension test using multiple choice questions designed by an expert panel [40]. To guaranty word recognition as well as comprehension the examinees read aloud 18 medical terms and associate each term with another word similar in meaning. The English as well as the Spanish version of the test demonstrate high correlations to other health literacy indices, display high reliability values and are particularly suitable to screen individuals with low health literacy [40]. One instrument developed to measure *health and financial literacy* addresses the link between literacy and decision making in the context of health related and financial factors. It examines health literacy by using 9 items dealing with health knowledge regarding health insurance, burden of disease as well as medication skills [41]. The test to measure critical health competencies *(CHC-Test)* consists of 72 items presented in 4 scenarios dealing with skills such as the understanding of medical concepts, searching literature, basic statistics and the design of experiments and samples [42]. The bilingual health literacy assessment *(Talking Touchscreen)* focuses on building a novel item pool in accordance with items used in the Test of Functional Health Literacy in Adults *(TOFHLA).* It measures prose, document and quantitative literacy in the field of certain lifestyle diseases as well as insurance related issues and patient rights administering these items with a multimedia gadget [43, 44]. A detailed description of the characteristics of instruments using an objective measurement approach is, presented in Table 2.

**Table 2:** **Main instrument characteristics categorized into objective, subjective and mixed measurement**

Instrument*	Author	Design and scope	Sample	Reliability	Validity sensitivity/Specifity	Instrument availability
***instruments with an objecitve measurement approach (N = 5)***						
**METER**	Rawson et al. 2009 [38]	40 medical words and 40 non-medical words Scoring: Low literacy (0–20); Marginal literacy (21–34) Functional literacy (35–40)	154 participants; mean age: 62.7 years (range: 29–88); 76.5% male; 92.6% white	Internal consistency: Cr. α = 0.93	REALM r = 0.74; 75% correct and 8% false positives identification	Available
**Talking Touchscreen**	Yost et al. 2009 [44]	Adoption of items from the NALS/NAAL framework and application to health-related materials; development of 138 items: 58 prose, 39 document, 41 quantitative Scoring: Not described	97 English participants, 134 Spanish participant; 65% female English, 74.6% female Spanish;	/	/	Partly available
**CHC Test**	Steckelberg et al. 2009 [42]	72 items; categories: Medical concepts, literature, statistics, design of experiments and sampling Scoring: not described	Phase 2: 322 trained and non-trained secondary school and university students; Phase 3: 107 grade 11 secondary school classes students	Phase 2: Reliability Rasch model = 0.88 Phase 3: Reliability Rasch model = 0.91	Effect size: Cohen’s d = 4.33	Not available
**SAHL-S&E**	Lee et al. 2010 [40]	32 items, reading test in Spanish and English Scoring: Cutoff point for low HL: ≤14	202 English-speaking and 201 Spanish-speaking participants aged 18–80 years	SAHL-S = 0.80 SAHL-E = 0.89	SAHL-S and SAHLSA: r = 0.88 SAHL-S and TOFHLA: r = 0.62 SAHL-E and REALM: r = 0.94 SAHL-E and TOFHLA: r = 0.68	Available
**Health and financial literacy**	James et al. 2012 [41]	9 questions in health literacy, 23 questions on financial literacy Scoring: Percentage correct out of total items (range 0–1)	525 participants mean age 82.6 years 76% female; 91.2% white	Internal consistency: Cr. α = 0.77	/	Available
***instruments with an subjecitve measurement approach (N = 5)***						
**MHLS-50**	Tsai et al. 2011 [48]	63 items with four sections: health materials, outpatient dialogues, prescription labels, health-related written documents Scoring: (0–30) inadequate health literacy; (31–42) marginal health literacy; (43–50) adequate health literacy	323 individuals; mean age = 47 years	Internal consistency: Cr. α = 0.95; Split half reliability = 0.95	Years of schooling r = 0.72 Reading habit r = 0.34 Health knowledge r = 0.55 Reading assistance r = -0.52	Not available
**HLS-CH**	Wang et al. 2012 [45]	Questionnaire of 158 items; 127 questions on 30 competencies for health Scoring: not described	1255 participants: (652 German-speaking, 303 French-speaking, 300 Italian-speaking) age +15 years	Internal consistency: Cr. α for each factor: Information and decision making α = 0.72, Cognitive and inter-personal skills α =0.81, ICT skills α = 0.77; Health activation α = 0.60	Correlations: Correlations: Cognitive and interpersonal skills and ICT skills factors = 0.50; Information and decision- making and ICT skills factors = 0.27	Not available
**AAHLS**	Chinn et al. 2012 [47]	4 items functional health literacy, 3 items on communicative health literacy, 4 items on critical health literacy, 3 empowerment items Scoring: not described	146 participants: mean age 38 years, 78% female; 56% Asian, 3% Black, 35% White	Internal consistency: Total items Cr. α = 0.75; Functional items Cr. α = 0.82; Communicative items Cr. α = 0.69; Critical items Cr. α = 0.42	Correlations: Functional & communicative items r = 0.393; Functional & critical items r = 0.59; Communicative & critical items r = 0.186	Partly available
**HELMS**	Jordan et al. 2013 [16]	8 domains with 29 items; capacity to seek, understand and use health information within the health care setting Scoring: not described	15 participants: 2 aged 40–49, 1 aged 50–59, 6 aged 60–69, 5 aged 70–79, and 1 aged 80+ years; 80% female	Test-retest: ICC = 0.73-0.96 (5 domains ICC > 0.90); Understanding health information: reliability = 0.73; Cr. α >0.82 for all factors	/	Not available
**MAHL**	Massey et al. 2013 [37]	Questionnaire, sixth grade reading level; adaption of items from YAHCS, HINTS and eHEALS Scoring: not described	1208 adolescents: mean age 14.8 years (range 13–17); 62.4% female; 22.1% white, 13.2% black, 33.7% Hispanic, 7.9% Asian	Internal consistency: all but one domain had Cr. α >0.7; overall = 0.834; lowest = 0.64	Consistency: average inter-item correlations (0.33 to 0.66); discriminability: item-total correlations (0.39 to 0.74)	available
***instruments with an mixed measurement approach (N = 7)***						
**HLSI**	McCormack et al. 2010 [36]	25 item instrument; skills set areas: print, oral, and Internet-based information seeking Scoring: ≥82: Proficient literacy; 70–81: Basic literacy; <70: Below basic literacy	889 participants; 22% 18–29 years, 25% 30–44 years, 27% 45–59 years, 26% 65+ years; 52% female; 64% white, 13% black, 17% Hispanic	Internal consistency: Cr. α =0.86	S-TOFHLA and HSLI correlation = 0.47; Sensitivity = 0.71; Specificity = 0.65	Available
**Canadian exploratory study**	Begoray et al. 2012 [52]	Qualitative open-ended questions; Questions on 2 reading passages Scoring: not described	229 participants; mean age 76 years (range 60–96); 65% female; 64%	Internal consistency: Cr. α =0.852; removal of any of the measures form the analysis reduced Cr. α down to 0.832	Reading passages scores & correlated REALM scores: spearman´s rho = 0.212; sum scale scores & English as first language rho = 0.228; sum scale scores & age rho = -0.176; education rho = 0.175 household income rho = 0.162	Partly available
**HL of Canadian high school students**	Wu et al. 2010 [53]	11 passages and 47items (30 understand and 17 evaluate items) Scoring: not described	275 students: 8% male; 69.1% speak a language other than English at home	Internal consistency: understand: Cr. α = 0.88; evaluate: Cr. α = 0.82; overall: Cr. α = 0.92	bivariate correlations: overall & age r = -0.173overall & gender r = -0.182 overall & GPA: r = 0.475 understand & evaluate: r = 0.80 understand & overall r = 0.97 evaluate & overall r = 0.92	Not available
**SLS and SNS**	McNaughton et al. 2011 [56]	SLS: 3 questions, each with a five-point likert response scale SNS: 8 written questions, each on a six-point likert response scale Scoring: not described	207 patients mean age 46 years (32–59) 55% male	Internal consistency: SLS: Cr. α = 0.74; SNS: Cr. α = 0.82	spearman´s rank: SLS and STOFHLA = 0.33 SLS and REALM = 0.26 SLS and WRAT4 = 0.26 SLS and educational = 0.25 AU ROC: SLS and STOFHLA AUC = 0.74 SLS and REALM AUC = 0.72	Not available
**SDPI-HH HL**	Brega et al. 2012 [59]	The questionnaire assesses 4 types of knowledge: general diabetes, insulin use, cholesterol, and blood pressure knowledge Scoring: Scores on each test reflect the percentage of items answered correctly	3,033 participants 5.9% aged 18–34, 15.5% aged 35–44, 28.2% aged 45–54, 30.4% aged 55–64, 20% aged 65+ years; 66.4% female	Internal consistency: PL items Cr. α = 0.67	/	Available
**HLSI-SF**	Bann et al. 2012 [50]	10 item instrument that measures print literay, numeracy, oral literacy, navigation through the internet Scoring: Number of items answered correctly	889 participants: 22% 18–29 years, 25% 30–44 years, 27% 45–59 years, 26% 60+ years; 52% female; 64% white, 13% black, 17% Hispanic	Internal consistency: Cr. α = 0.70	Correlation with S-TOFHLA r = 0.36	Available
**HLS-EU**	HLS-EU Consortium 2012 [8]	47 items; in three domains: health care, disease prevention, health promotion Scoring: Metric between 0-50	8102 participants from Germany, Greece, Bulgaria, Ireland, Austria, Spain, Netherlands, Poland	Internal consistency Cr. α: Gen HL = 0.97 HC HL = 0.91 DP HL = 0.91 HP HL = 0.92	/	Partly available

*MHLS-50 = Mandarin Health Literacy Scale; HLS-CH = Swiss Health Literacy Survey; AAHLS = All Aspects of Health Literacy Scale; HeLMS = Health Literacy Management Scale.

*MAHL = Multidimensional measure of adolescent health literacy; HLSI = skill-based health literacy instrument.

*SAHL-S&E = Short assessment of health literacy – Spanish and English; SDPI-HH-HL: Special Diabetes Program for Indians Healthy Heart Health Literacy; HLSI-SF = Health Literacy Skills Instruments – Short Form; HLS –EU = Health Literacy Survey for the European Union.

### Health literacy assessment by subjective measurement tools

All identified instruments measuring health literacy by a self-report use a multidimensional concept of health literacy by integrating several domains and factors associated with health literacy. The self-report approach was applied in five instruments published in the search period. The Multidimensional Measure of Adolescent Health Literacy *(MAHL)* assesses health literacy as a dynamic construct by addressing several domains: patient-provider encounter, interaction with the health care system, rights and responsibilities and health information. These are developed by analyzing items of numerous already existing instruments, identifying relevant items and modifying as well as supplementing them by new items [37]. The Health Literacy Management Scale *(HELMS)* consists of 8 scales with 4–5 items and aims to assess health literacy by using a comprehensive approach. It encompasses multiple domains such as patient attitudes towards health and their proactivity as well as access, understanding and use of health information and access and communication with healthcare professionals [16]. The 127 item Swiss Health Literacy Survey *(HLS-CH)* also addresses numerous domains such as information and (critical) decision making, cognitive and interpersonal skills as well as problem solving. In this regard health literacy is rather a package of competencies interacting with each other [45]. The All Aspects of Health Literacy *(AAHLS)* measures health literacy based on the framework developed by Nutbeam [46] and measures functional, communicative and critical literacy by using 14 items derived from an analysis of already existing scales in the field of health as well as media literacy [47]. Seemingly relevant items from numerous sources were adopted, partially modified, and supplemented resulting in an adequate overall reliability of Cronbach’s alpha = 0.74 whilst weak consistency among the subscales. The 63 item Health Literacy Scale developed in Taiwan *(MHLS)* also captures health literacy as a multi-domain construct encompassing obtaining, understanding and processing health related information related to health promotion, disease symptoms, diagnosis, and treatment and using them in decision making [48]. A further detailed description of the characteristics of instruments applying a subjective measurement approach is, presented in Table 2.

### Health literacy assessment by a mixed measurement approach

The combination of a direct testing and a self-report of health literacy skills is practiced frequently among indices, thus seven instruments identified in the search period use this approach. It enables to combine the methodological advantages of both approaches by diminishing possible straits [49]. The Health Literacy Skills instrument *(HLSI)* as well as the short form *(HLSI-SF)* are 25/10 item tools that use real life health stimuli to assess an individual’s health literacy addressing print, oral, quantitative and internet based information seeking skills. The short form is derived by analyzing the psychometric properties of the HLSI and selecting best performing items. Additionally an 8 item self-report of the perceived performance among the skills addressed in the direct assessment of health literacy is conducted. Both approaches assess print literacy, numeracy and oral literacy as well as media literacy in a different manner demonstrating an acceptable internal consistency reliability of a Cronbach’s alpha of 0.86 for the HLSI and 0.70 for the HLSI-SF [36, 50]. The European Health Literacy Survey *(HLS-EU)* carried out in eight European countries (Germany (NRW), Bulgaria, Austria, Greece, Spain, Ireland, Netherlands, Poland) also uses a mixed assessment approach measuring functional health literacy with the Newest Vital Sign (*NVS)* and using a self-report survey with 47 items. It defines health literacy in three domains (health care, disease prevention, health promotion) and 4 modes (access, understand, evaluate and apply health information). Though the HLS-EU demonstrates a robust reliability of a Cronbach’s alpha of 0.97 for general health literacy the Spearman’s rho correlation between the NVS and HLS-EU with r = .245 is comparatively low indicating different constructs of health literacy [8, 49, 51]. Similar findings are apparent in the Canadian explanatory study aiming to define a health literacy measure by combining nine self-report items dealing with the access, understanding and appraisal of health information as well as communication skills in the patient provider encounter. Additionally, nine task performance (objective) items focus on understanding health related skills. A correlation between the measurement approaches could not be demonstrated [52]. A further Canadian study developing an instrument for measuring the health literacy of Canadian high school students focuses on skills to understand and evaluate health information. It uses 11 health related passages from several sources (internet, heath centers, health education and media materials) and develops 47 items examining the comprehension and interpretation of the presented information in the passages. A self-rating of health literacy skills is also included. Despite of a satisfactory overall reliability value of a Cronbach’s alpha of 0.92, bivariate correlations of r = 0.256 between the self-rating and the direct testing doesn’t indicate a strong coherence [53]. The brief subjective measure of numeracy (SNS) and general health literacy (SLS) is an 11 item instrument combining a subjective measurement of functional literacy by using the SBSQ [54] and the subjective numeracy scale (SNS) [55] with numerous previously developed objective indices to scale down bias of self-reports demonstrating a robust internal reliability [56]. The health literacy measurement applied in the special diabetes program for Indians (SDPI-HH-PL) follows a similar approach by combining items of the SBSQ to measure document literacy by a self-report and items of previously published instruments to measure numeracy by directly testing quantitative skills [54, 57–59]. Though the mixed measurement approach broadens the health literacy framework some studies indicate an absence of coherence between the underlying constructs subsequently detecting missing correlations between the measurement approaches [8, 52, 53]. A further detailed description of the characteristics of instruments applying a mixed measurement approach is, presented in Table 2.

### Reporting quality of identified health literacy instrument studies

The application of reporting guidelines is a useful way to facilitate transparency and gauge the reliability of an instrument used in a survey. However the compliance with reporting guidelines such as the “reporting guideline for survey research” recently compiled by Bennett and colleges [21] is limited among papers reporting on the development and validation of health literacy indices as depicted in Table 3. Among the 17 identified publications, about a third does not report on the significant reporting features specified in the guideline. The reporting frequency varies across different domains of the guideline. Study objectives, presentation of the results as well as interpretation and discussion of the findings are appropriately described in all publications. Article parts related to methodological issues such as data replication and verification (58,8%), the procedures of sample selection such as sample size calculation (23,5%), and representativeness of the sample (41,2%) are reported noticeably less as described in Table 3. Furthermore, the description of the characteristics of health literacy indices is limited among features such as the instrument pretesting, reported reliability and validity as well as the scoring method, not described in 52,9%, 23,5% and 64,7%, respectively, of all publications. Additionally, 58,8% (n = 10) of the articles do not present items of the instrument entirely making it difficult to perform an appraisal as presented in Table 2. Though reflection of non-response is central among the analysis of quantitative data, only two third of the publications do report these and 82,4% do not discuss the role of non-response rates among the performed study as listed in Table 3. Similar findings apply to the handling of missings, which are not described in more than two third of the publications. However several checklists provide guidance on the reporting of survey research and instrument development and could be used in order to report on study results adequately [60, 61].

**Table 3:** **Survey reporting quality of identified studies dealing with the development and/or validation of health literacy indices**

Checklist items	Fully described		Not described	
**Background**	**N**	**%**	**N**	**%**
Background literature review	16	94,1	1	5,9
Explicit research question	16	94,1	1	5,9
Clear Study objectives	17	100,0	0	0,0
**Methods**				
Methods data analysis	16	94,1	1	5,9
Questionnaire administration	14	82,4	3	17,6
Location of data collection	17	100,0	0	0,0
Dates of data collection	8	47,1	9	52,9
Methods for replication	10	58,8	7	41,2
Methods for data entry	10	58,8	7	41,2
**Sample selection**				
Sample size calculation	4	23,5	13	76,5
Representativeness of the sample	7	41,2	10	58,8
Method of sample selection	17	100,0	0	0,0
Population and sample frame	15	88,2	2	11,8
**Research tool**				
Description of research tool	15	88,2	2	11,8
Development of research tool	16	94,1	1	5,9
Instrument pretesting	8	47,1	9	52,9
Reliability and validity	13	76,5	4	23,5
Scoring methods	6	35,3	11	64,7
**Results**				
Results of research presented	17	100,0	0	0,0
Results address objectives	17	100,0	0	0,0
Generalisability	5	29,4	12	70,6
**Response rate**				
Response rate stated	11	64,7	6	35,3
Response rate calculated	7	41,2	10	58,8
Discussion of nonresponse	3	17,6	14	82,4
Missing data	6	35,3	11	64,7
**Interpretation and discussion**				
Interpret and discuss findings	17	100,0	0	0,0
Conclusions and recommendations	17	100,0	0	0,0
Limitations	14	82,4	3	17,6
**Ethics and disclosure**				
Consent	8	47,1	9	52,9
Sponsorship	8	47,1	9	52,9
**Mean reporting frequency**		69,7		30,4

## Discussion

In our review, we identified recently published (2009 forward) publications dealing with novel instruments developed and validated to measure health literacy. The review followed two main objectives. In the first place, we examined how the measurement of health literacy proceeded in recent years particularly emphasizing whether novel instruments consider existing recommendations of the scientific community on features an instrument measuring health literacy should cover. In addition, we analyzed the reporting quality of the identified papers dealing with the development of health literacy measurement tools.

Our analysis resulted in six major findings, which extend the prior knowledge on health literacy measurement.

First of all, we examined an increasing use of multidimensional constructs to measure health literacy. Especially instruments with a subjective measurement format address numerous domains of health literacy such as patient-provider encounter; interaction with the health care system; rights and responsibilities; health information-seeking; understanding, processing, and using healthcare information as well as communication with healthcare professionals [8, 16, 36, 37, 45, 48, 50]. In this regard, earlier critiques towards the one-dimensional measurement modes usually used in health literacy measurement are taken into consideration when developing novel instruments [12]. This in turn allows a more in depth and comprehensive operationalization of the dynamic construct “health literacy” and helps to improve the measurement.

Furthermore, we found that almost all instruments apply a multidimensional measurement of health literacy by principally assessing print literacy and numeracy and in some cases adding oral literacy. Previous reviews dealing with health literacy measurement tools emphasized the lack of instruments integrating communication skills (oral literacy) in the health literacy construct [17]. To fill this gap, three novel instruments containing oral literacy were developed and validated in the search period of our review (2009 forward) [16, 36, 50]. This result further indicates that newly developed instruments take the recommendations of the academic circle into consideration.

In addition we identified that there is a trend towards the use of objective (task based) and subjective (self-report based) measurement approaches in a mixed manner. Scholars using this mixed measurement approach often apply already existing health literacy screeners (e.g. SBSQ; NVS) and develop additional item batteries [8, 56, 59]. Principally the mixed measurement approach offers advantages by broadening the health literacy concept and enabling researchers to address multiple skills. However, studies using this approach in our review found a weakness of coherence between the underlying constructs measured by the different approaches. This subsequently results in limited correlation between the measurement approaches [8, 52, 53]. Consequently, these results should be taken into consideration when using the mixed measurement approach.

A further striking finding is that regardless of the used measurement approach, scholars do not sufficiently explain why they are choosing a certain type of measurement. According to Abel, the first issue in the circle of instrument development is to determine the purpose of the instrument by answering the “what for” question. As soon as the given theoretical context and setting is clear, ideas on the way of measurement can be developed systematically [62]. If the reason for a certain approach is not clearly determined, the development of a structured and comparable procedure to measure health literacy will be hard to achieve.

Finally, there is an extensive use of assessment formats modeled on already existing instruments such as the REALM or the TOFHLA inserting mostly straightforward additions [37, 38, 40, 44, 47]. Since these instruments have many weaknesses, researchers are calling for the development and use of new measurement approaches to avoid stagnation [17].

The appraisal of the reporting quality of publications dealing with the development and validation of health literacy indices has yielded mixed findings. Some domains such as the description of the article background and presentation and interpretation of results are reported thoroughly, while other domains addressing methodological properties have received less consideration. Overall, the identified papers included in the review demonstrate a lack of compliance with reporting guidelines especially for methodological issues such as psychometric properties of the developed instruments, sample selection strategy and response rate presentation. These findings are in line with previous research stating that key survey characteristics in health care literature in general [63, 64] and in health literacy research in particular [13] are often underreported. Although Jordan and colleges had already identified these weaknesses in their review considering measurement tools published between 1990 and 2008 [13] only few improvements are noticeable. Especially the reporting on the psychometric properties (reliability, validity) of the instruments is still not appropriate in nearly one third of all instruments. Additionally more than two third of the articles neither mention the issue of instrument scoring nor discuss the significance of non-response in the study setting. These findings demonstrate potential for further enhancements in improving health literacy research.

From an overall perspective, almost all identified instruments apply a multi-dimensional measurement (often print and numeracy literacy) and the majority utilizes a mixed measurement approach (objective and subjective measurement) with a multidimensional construct enhancing the comprehensiveness of tools measuring health literacy. Nevertheless, there is no clear indication of the demanded “consensus” on health literacy measurement. This is mainly because there have been only minor developments among the measurement formats, as can be seen in the increased use of earlier instruments, even though the academic world is calling for new instruments [17].

To continuously advance the field of health literacy measurement work should proceed on several fronts. Though there is currently a huge effort to improve the more comprehensive measurement of health literacy, the format of measurement generally relies on already existing approaches such as the cloze technique (used in the “The test of functional health literacy in adults” (TOFHLA)) or word recognition (used in “Rapid estimate of adult literacy in Medicine” (REALM)) [37, 38, 40, 44, 47]. Therefore, future health literacy research should strongly emphasize the development of new measurement approaches such as skill-based concepts with a generic approach [36, 50]. Here, the use of vignettes assessing ones abilities in a daily life setting could be an innovative step towards an approach that is already being used for measuring mental health literacy [65]. Consideration of measurement formats used in the field of information literacy could be also of great interest as they focus on the handling of information [66, 67]. Of course, these need to be tailored to the capacity of lay people.

Apart from the issue of originality, it would be necessary to reflect more closely on the combination of objective and subjective measurement instruments, thus current studies show less coherence. Though the limited reporting guideline compliance of health literacy instruments was identified by Jordan and colleges before [13] our analysis displays similar findings. Especially the poor reporting of the scoring methods and the weaknesses among the currently used procedures to determine construct validity need to be improved. Thus, construct validity is most often measured by comparing the instrument with screeners assessing functional literacy derived from standardized literacy tests without taking into account that health literacy is a dynamic and comprehensive construct and therefore not comparable with tests. The described procedure does not contribute to the qualitative improvement of health literacy indices but increases a path dependency. The consequences are recognizable among newly developed instruments in European countries often simply translating literacy based screeners developed in English speaking countries [32, 33] without considering cultural and institutional differences.

In considering such recommendations, certain limitations should be noticed regarding our review. Although we followed the PRISMA guidelines when performing our systematic review and used MESH terms and key words, we may have missed relevant literature. Furthermore, there was no reporting guideline available that provided a scoring scheme for the reporting quality. As a consequence we could not grade the reporting quality of the identified articles resulting in a descriptive description of the results. Finally, the appraisal of health literacy instruments was limited as the item batteries and scoring methods were not always available despite a direct request to the authors.

Apart from this, our review exhibits certain strengths such as the compliance to guidelines when performing the literature search, data selection, analysis and appraisal of the reporting quality of the identified articles.

## Conclusions

Our review offers insights in the status quo of health literacy measurement. It critically appraises applied measurement approaches and analyses reporting qualities by commenting on current developments and their value for the further evolution of health literacy measurement. Giving attention to the evidence presented here can help to offer direction towards the development of comparable and reliable health literacy assessment tools that effectively respond to the informational needs of populations.

## Authors’ information

All authors are affiliated to the Institute for Health Economics and Clinical Epidemiology, University Hospital of Cologne. and primarily deal with health systems and outcomes research focusing on chronic care and disease management. Mrs. Prof. Dr. med. Stephanie Stock is the chairwoman of the German Health Literacy Network and coordinates the network activities in Germany.

## Electronic supplementary material

Additional file 1: **Prisma Checklist.** (DOC 66 KB)

Additional file 2: **Overview of the chosen search strategy.** (DOCX 12 KB)
