# Supplementary material for: The evolution of health literacy assessment tools: a systematic review
Source: BMC Public Health. 2014 Nov 24;14:1207. doi: 10.1186/1471-2458-14-1207 (PMC4289240; doi:10.1186/1471-2458-14-1207)
Supplement: Supplementary file 2 — Additional file 2: Overview of the chosen search strategy. (DOCX 12 KB) [file 12889_2014_7389_MOESM2_ESM.docx]

Additional file 1:

Appendix 1: Overview of the chosen search strategy

| **Database** | **Search strategy** |
| --- | --- |
| PubMed | (Health Literacy[MeSH] OR "Health Literacy") AND (Educational Measurement[MeSH] OR Test Taking Skills[MeSH] OR Nursing Evaluation Research[MeSH] OR (Assessment tool OR assessment tools) OR Measurement OR (Measurement tool OR measurement tools) OR Assessment) |
| Cumulative Index to Nursing and Allied Health Literature  (CINAHL) | (TX Health Literacy) AND TX (Assessment tool OR instrument)  Subject: Major Heading  (health knowledge, literacy, patient education, instrument validation, medication compliance, information literacy, health behavior, health education, self-care, patient attitudes, health services accessibility, instrument construction, consumer health information, attitude to health, health information, health promotion, information needs, physician-patient relations, teaching materials, nurse attitudes, patient compliance, questionnaires, communication barriers) |
| Educational Resources Information Center (ERIC) | “health literacy” |
| Web of Knowledge (WOK) | “health literacy” AND (assessment tool OR measurement) |
